# Steroid Hormones and Their Action in Women's Brains: The Importance of Hormonal Balance

**DOI:** 10.3389/fpubh.2018.00141

**Published:** 2018-05-23

**Authors:** Juan Pablo Del Río, María I. Alliende, Natalia Molina, Felipe G. Serrano, Santiago Molina, Pilar Vigil

**Affiliations:** ^1^Reproductive Health Research Institute Santiago, Chile; ^2^Tallahassee Community College Tallahassee, FL, United States; ^3^Vicerrectoría de Comunicaciones Pontificia Universidad Católica de Chile, Santiago, Chile

**Keywords:** estrogens, progesterone, hormones, neurosteroids, ovarian continuum, neurotransmitters, neuroprotection

## Abstract

Sex hormones significantly impact women's lives. Throughout the different stages of life, from menarche to menopause and all stages in between, women experience dramatic fluctuations in the levels of progesterone and estradiol, among other hormones. These fluctuations affect the body as a whole, including the central nervous system (CNS). In the CNS, sex hormones act via steroid receptors. They also have an effect on different neurotransmitters such as GABA, serotonin, dopamine, and glutamate. Additionally, studies show that sex hormones and their metabolites influence brain areas that regulate mood, behavior, and cognitive abilities. This review emphasizes the benefits a proper hormonal balance during the different stages of life has in the CNS. To achieve this goal, it is essential that hormone levels are evaluated considering a woman's age and ovulatory status, so that a correct diagnosis and treatment can be made. Knowledge of steroid hormone activity in the brain will give women and health providers an important tool for improving their health and well-being.

## Introduction

Levels of sex hormones, such as estrogen and progesterone, fluctuate naturally throughout the different stages of a woman's life (1). In women, steroid hormones are mostly synthesized in peripheral glands and the adipose tissue as well as in the brain (2). Estrogen and progesterone act by binding to steroid receptors through the classical pathway, making use of intracellular receptors that, after a series of conformational changes, find their way into the nucleus where they regulate gene expression (3). Additionally, sex steroid receptors can be found outside the nucleus, including mitochondria, the endoplasmic reticulum, and the plasma membrane, where they activate different signaling cascades exerting their action through a non-classical pathway (2).

Steroid hormones with activity in the nervous system are called “neurosteroids” or “neuroactive steroids” (2, 4, 5). They may be synthesized de novo in the central and peripheral nervous systems by neurons and glial cells or, peripherally and then cross the blood-brain barrier (6). Although it has been shown that levels of steroid hormones in peripheral blood differ from those obtained in cerebrospinal fluid, measurement of their plasma levels will be important for the understanding of brain function, since steroid hormones cross the blood-brain barrier. It is possible to classify the effects of these hormones on the CNS as activational or organizational (7). Activational effects modify neural activity in a specific context and in a non-permanent manner through classical and non-classical pathways; for example: modulating glutamatergic, GABAergic, serotonergic, and dopaminergic synapses (2, 8). Organizational effects include the capacity that molecules, such as sex steroids, have to permanently alter the structure of the nervous system through a variety of mechanisms, such as myelination, neural pruning, apoptosis, and dendritic spine remodeling (9–11).

A good example of these effects is the role that neurosteroids have in modulating synaptic plasticity through long-term potentiation (LTP). This neuromodulative process refers to events that produce an increase in synaptic strength, which persists in time and correlates with the processes of memory and learning (12, 13). LTP can be observed in the hippocampus, a region that plays an important role in the consolidation of information from short-term memory to long-term memory and in spatial memory, where estrogen generates changes in plasticity and induces an improvement in cognitive functions (14–17).

Through their organizational and activational actions in the CNS, neurosteroids regulate different brain areas involved in the modulation of mood, behavior, and cognition (11, 18). Therefore, the endogenous sexual hormonal fluctuations during specific reproductive stages of a woman's life are related to an increased susceptibility of women to develop mood disorders, as premenstrual dysphoric disorder, postpartum depression, and perimenopausal depression (11, 19, 20). In addition, endogenous estrogen and progesterone levels also may affect different cognitive processes such as decision-making, emotion recognition, consolidation of emotional memory, and fear extinction (21, 22). For example, during the menstrual cycle, women show improved verbal abilities and decreased visual-spatial abilities when estradiol and progesterone levels are high, however, when estradiol and progesterone levels are low the opposite is observed (23). Also, low levels of estradiol and progesterone (i.e., in ovariectomized non-human primates) induce spatial memory deficits, which are reversed with cyclical, low-dose estrogen treatment (24). This is consistent with recent studies that show that neurosteroids could be an effective therapeutic strategy against psychiatric disorders, such as schizophrenia, depression, and also against neurodegenerative disorders, such as Alzheimer's, Parkinson's, and multiple sclerosis (6, 25).

In the present review we analyze steroid hormone production throughout a woman's life and the mechanisms through which neurosteroids affect neural cells in the female brain. We propose that a hormonal balance of sex steroids proper to the different life stages will help improve women's well-being throughout their lives and prevent neurocognitive dysfunctions.

## Hormonal production throughout a woman's life

### The ovarian continuum

**Figure 1:**
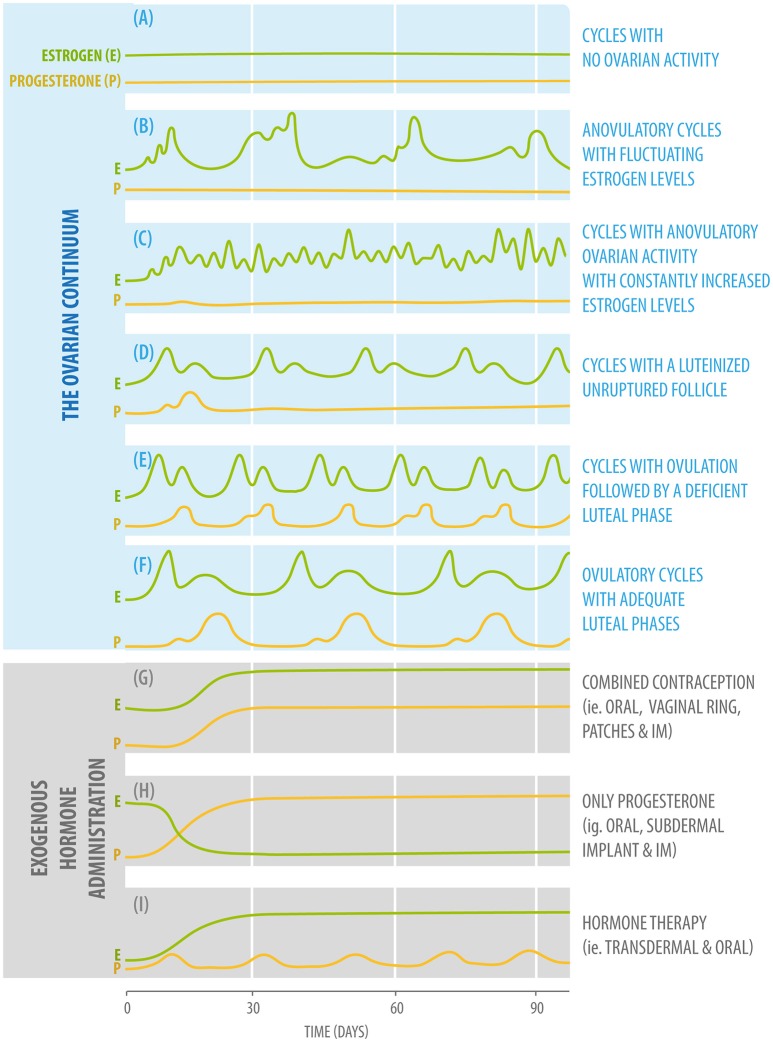
Diagram of fluctuating plasma estradiol (green) and progesterone (yellow) values according to the different phases of the ovarian continuum (upper: **A–F**,) and three different forms of exogenous hormone administration (bottom: **G–I**).

The ovarian continuum can be understood as the various types of ovarian activity that a woman can present throughout her lifetime, starting in intrauterine life (1, 26). When considering the ovarian continuum, a healthy child is in an anovulatory state, with low plasma estradiol values (27). During puberty, gonadotropin levels start to rise, which causes an increase in estradiol plasma values (28). Menarche usually indicates that the first ovulation has occurred (29). Once the reproductive system fully matures, women between 12 and 50 years of age normally exhibit regular ovulations characterized by 24- to 36-day cycles with fluctuating plasma estradiol and progesterone values according to the different phases of the cycle (Figure 1A) (Box 1).

Box 1 **Main hormonal steps during a normal ovulatory cycle (26).**

1. Follicle stimulating hormone (FSH) level increase leads to recruitment and development of ovarian follicles.

2. Selected follicles produce rising estradiol levels.

3. Estradiol, together with inhibin, exerts a negative feedback upon the hypothalamic-pituitary-gonadal (HPG) axis decreasing FSH levels. Estradiol also has a negative feedback effect on the kisspeptinergic neurons (30).

4. One of the selected follicles becomes the dominant follicle.

5. Estradiol levels, produced by the dominant follicle, rise to peak levels and, together with a small rise in progesterone, exert a positive feedback on the kisspeptinergic neurons, hypothalamus, and hypophysis.

6. The positive feedback mechanism induces the mid-cycle gonadotropin surge.

7. Follicular rupture and ovulation occur.

8. Progesterone rises due to follicular luteinization and the corpus luteum is formed.

9. The corpus luteum secretes progesterone and estrogens, which further inhibits follicular development.

10. If fertilization does not occur, the corpus luteum will last for 11 to 17 days.

11. Estradiol and progesterone concentrations drop, eliminating the negative feedback exerted upon the HPG axis.

12. A new cycle begins.

The functional capacity of the ovary diminishes with age. Approximately 4 years before their final menstrual period (menopause), women enter the perimenopausal period (31) characterized by symptoms such as headaches, sleep disturbances, mood fluctuations, anxiety, depressive symptoms, impairment in cognitive function, hot flashes and, later on, vaginal dryness and bone fractures, among others (32, 33). During this period, due to gonadotropin stimulation, increased estrogen levels are produced by the ovaries, which cause endometrial growth that can be associated with heavy bleeding and irregular cycles (31).

Hormonal levels will reach a state of little or no fluctuation approximately 2 years after menopause, when estradiol and progesterone values remain low (Figure 1A), while FSH levels remain high (34). A decrease in steroid hormones has been associated with cognitive impairments, reflected in a higher incidence of neurodegenerative diseases such as Alzheimer's disease (35).

It is important to consider the effects that the aging process has on the reproductive axis, particularly at the CNS level (36). With age, the hypothalamic GnRH neurons, that contribute to regulate the cyclicity of the menstrual cycle, are affected in a functional and morphological manner (36). For this reason, it is not possible to completely isolate the aging phenomena of the endocrine system from the aging processes that occur in the nervous system.

### Phases of the ovarian continuum

According to the concept of the ovarian continuum (1), cycles can be classified as follows:

Cycles with no ovarian activity, in which estradiol and progesterone plasma levels are low. This type of ovarian activity will be present in childhood and menopause. It can also be found in cases such as anorexia and hypogonadotropic hypogonadism (Figure 1A).

Anovulatory cycles with fluctuating estrogen levels. Estrogen levels will start to rise, but will not be adequate to induce a luteinizing hormone (LH) rise and ovulation. This type of ovarian activity will be found during pubertal development and, for example, in women who are partially breastfeeding (Figure 1B).

#### Cycles with anovulatory ovarian activity with constantly increased estrogen levels

This pattern of ovarian activity can be found in women with Polycystic Ovarian Syndrome, usually associated with the presence of increased adiposity because adipocytes can produce estradiol in considerable amounts (Figure 1C).

#### Cycles with a luteinized unruptured follicle

This type of ovarian activity can be found during the perimenopausal period and in cases of hyperprolactinemia (Figure 1D).

#### Cycles with ovulation followed by a deficient luteal phase

As the previous type of activity, this pattern can also be found after breastfeeding during the period of returning fertility, the menopausal transition, and in women presenting hypothyroidism and hyperprolactinemia (Figure 1E).

#### Ovulatory cycles with adequate luteal phases

This is the type of ovarian activity present when an adequate hormonal balance between estradiol and progesterone is found (Figure 1F) (26).

When the hormonal balance between estrogen and progesterone is disrupted, women are at greater risk of experiencing neurocognitive dysfunctions (5). For this reason, whenever a woman is being treated for a mental health condition, her pattern of ovarian activity should be taken into consideration. Some of the mechanisms that may help to elucidate why this association occurs are described below.

## Hormone actions in the female brain

### Classical and non-classical pathways of estradiol and progesterone action

#### Estradiol

In the classical estrogenic pathway, estrogens diffuse into target cells activating estrogen receptors (ER) α and β which form dimers. The activated receptors go into the nucleus, where they bind to estrogen responsive elements (ERE) of DNA (37, 38). It has been shown that ERα and ERβ are also located in mitochondria, where they bind to mitochondrial DNA (39, 40). The activation of the EREs results in gene transcription at the nuclear and mitochondrial levels.

In the non-classical pathway, estrogens act through different receptors (ERα, ERβ, GPER, and GqmER) located in the plasma membrane (41–43). Through these receptors, estrogen triggers the activation of different signaling cascades such as phosphatidylinositol-3-kinase (PI3K), phospholipase C (PLC), and mitogen-activated protein kinases (MAPK), second messengers, ion influx, and efflux. Finally, genomic transcription can also be induced by the non-classical pathway (43).

The activation of both pathways via estrogenic action will exert a neuroprotective effect in the CNS through different mechanisms:

- Activation of anti-apoptotic and cell survival pathways: this action considers the expression of the transcription factor CREB (cAMP response element-binding), which in turn upregulates the transcription of neuronal survival and neurotrophic genes, such as BDNF (brain-derived neurotrophic factor) (40, 43). Estrogen action also enhances the transcription of anti-apoptotic genes such as BCL2 (B-cell lymphoma 2) (44). Among its functions, BCL2 protects cells from excess of intracellular calcium by promoting its mitochondrial uptake and preventing the activation of different calcium-dependent enzymes that would damage cell structures (45).
- Additionally, through activation of PI3K and MAPK pathways, estrogen provides a coordinated response that results in the inactivation of the pro-apoptotic protein BAD (BCL-associated death promoter) (45, 46). Yet another mechanism by which estrogen enhances neuronal survival occurs through its activity in mitochondrial DNA, where it enhances the expression of enzymes that reduce free radicals, diminishing oxidative damage, and its consequential apoptotic process (47).
- Regulation of bioenergetic systems: estrogen regulates metabolic functions and sustains the energetic demands of neural cells by increasing the availability of glucose within the cell and ATP production by mitochondria (47). These mechanisms are possible due to the increase in the number of glucose transporters, glucose uptake, and the activity of glycolytic enzymes in aerobic glycolysis (40). Estrogen also increases the transcription of proteins and metabolic enzymes (i.e., pyruvate dehydrogenase, aconitase, and ATP synthase) needed for the generation of ATP (48).
- Mitochondrial activity is also promoted through an increase in calcium influx by the enhancement of the transcription of BCL2 gene. In this way, this gene not only has a neuroprotective effect, as explained above, but also contributes to regulate bioenergetic systems (43, 45).
- Regulation of neurogenesis: estrogen induces a significant proliferation of neural progenitor cells in a time- and dose-dependent manner (14, 40). It has been shown that this action is due to a rise in MAPK which in turn increases the neurotrophic factor BDNF, which protects neurons from degeneration (14, 49, 50).

#### Progesterone

In the classical pathway, progesterone diffuses into the cell and binds to its receptors (PRα and PRβ), acting through specific progesterone response elements (PREs) within the promoter region of target genes, and thus regulates transcription. Progesterone, and some of its neuroactive metabolites, such as allopregnanolone and dihydroprogesterone (DHP), also act through the non-classical pathway. This pathway includes membrane receptors such as PRα, PRβ, PQMR, and PGMR1 (51, 52). Through these receptors, progesterone triggers the activation of different signaling cascades [PI3K, PKC MAPK, protein kinase A (PKA)], second messengers, ion influx and efflux, and finally, the transcription of different genes (53).

Both the classical and non-classical pathways of progesterone have neuroprotective effects in the CNS causing: (i) a rise in anti-apoptotic mechanisms and cell survival, (ii) regulation of the bioenergetic systems (47), and (iii) induction of neural cell proliferation more consistently than estrogen (54). It must be emphasized that, although, estrogen and progesterone are potent regulators of cell survival, bioenergetic systems, and neurogenesis; the combination of estrogen and progesterone is not synergistic and, when administered in combination, at the same time, leads to a lower response compared to either hormone administered alone or in sequence (47, 53).

Another neuroprotective role of progesterone is its regulatory effect upon glial cells, where myelination has special relevance (55). In the peripheral nervous system myelin is synthesized by Schwann cells, while oligodendrocytes accomplish this in the CNS (56). In the CNS, one oligodendrocyte can extend up to 40 processes onto multiple adjacent axons, thus, each oligodendrocyte influences the electrical activity of a large number of axons (57).

**Figure 2:**
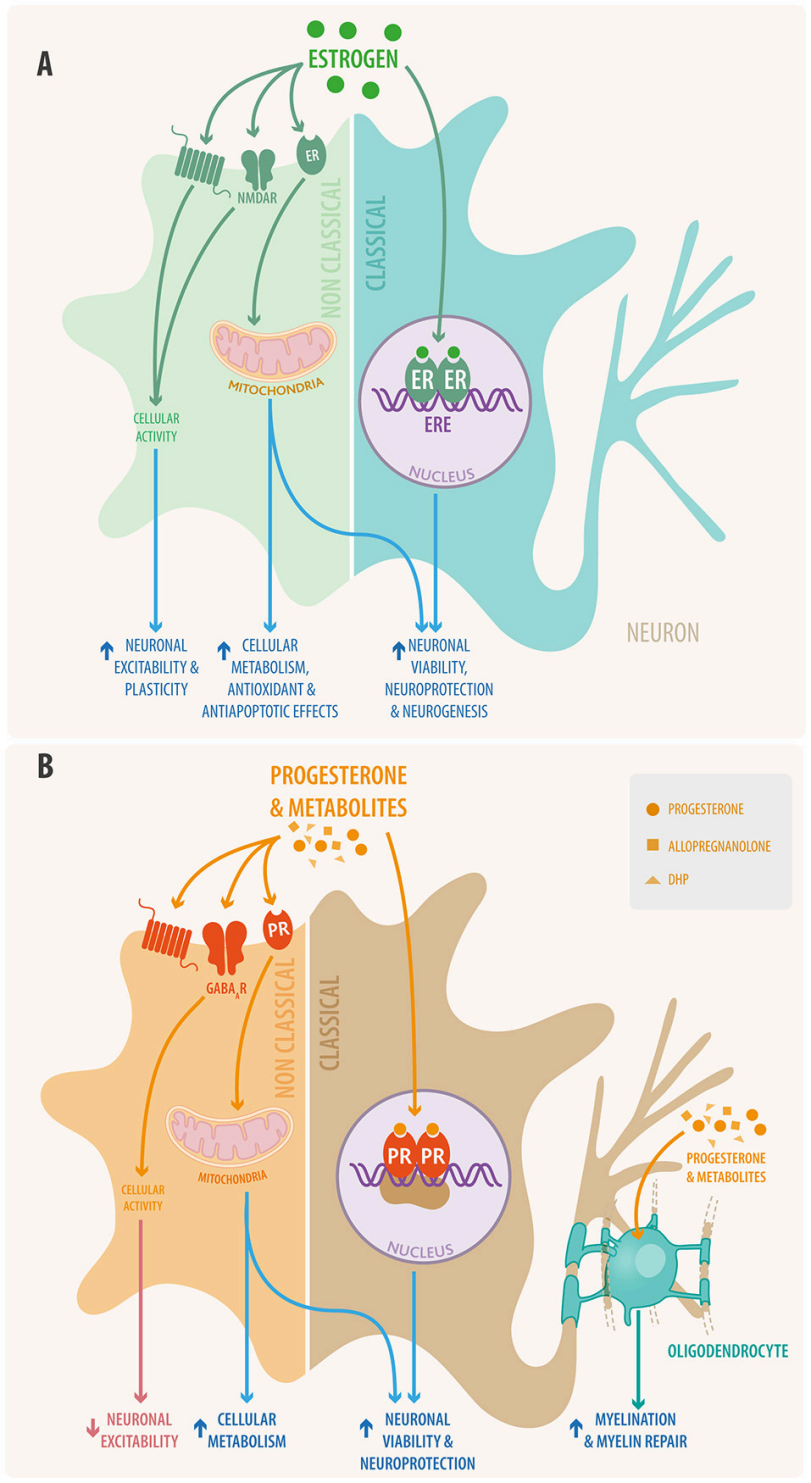
Schematic view of **(A)** estradiol and **(B)** progesterone signaling in neural cells through both classical and non-classical pathways. In the classical signaling pathway (right) the steroid hormone binds to its receptor located in the cytoplasm; the activated receptor dimerizes and makes its way into the nucleus where it interacts with responsive elements to activate or inhibit gene transcription. Also steroid hormones can bind to mitochondrial receptors that regulate mitochondrial DNA transcription. In the non-classical pathway (left) steroid hormones act through membrane receptors, including the classical receptors, GPCR receptors, ionotropic receptors, tyrosine kinase receptors, and other neurotransmitter receptors. This non-classical pathway initiates cytosolic signaling cascades, modulating the activation of various proteins and of second messenger systems. Additionally, progesterone and its metabolites promote myelination and remyelination at the oligodendrocyte level in the CNS and that of Schwann cells in the PNS.

Oligodendrocytes can proliferate from oligodendrocyte progenitor cells (OPC), which migrate toward unmyelinated axons, where they mature and form processes able to form the myelin sheath (57). Progesterone plays an important role in the stimulation of these steps (58) by promoting intracellular signaling, proliferation of oligodendrocyte progenitors, and transcription of key components in the synthesis pathways of myelin (i.e., myelin basic protein and 2', 3'-cyclic nucleotide-3'-phosphodiesterase) (57–59). Oligodendrocytes and their precursors produce high amounts of progesterone and metabolize progesterone from the bloodstream, or other sources, into DHP and allopregnanolone. It has been shown that, like progesterone, DHP also plays a role in the regulation of oligodendrocyte function and myelination (57). Furthermore, allopregnanolone acts like a potent positive allosteric modulator of GABA-A receptors. This modulation induces the proliferation of OPC in the form of an autocrine/paracrine loop (60). Therefore, progesterone and its metabolites have an important role in promoting cellular maturation and oligodendrocyte function in the CNS (Figure 2).

In the PNS, neurons and Schwann cells produce progesterone and, as oligodendrocytes do in the CNS, metabolize it to DHP and allopregnanolone. Through the classical pathway, via PR, progesterone and DHP bind to myelin gene promoters P0 and P1, which express myelin sheath specific proteins [i.e., glycoprotein P0 (P0) and peripheral myelin protein 22 (PMP22)] (61).

Allopregnanolone, via GABA receptors, promotes the production of GABA, which induces the proliferation of Schwann cells (61, 62). Through these mechanisms, progesterone and its metabolites modulate the myelination and remyelination processes in the PNS. It is important to mention that some of these actions could explain the fundamental role of progestogens in myelin repair under neurodegenerative conditions (63).

### Estradiol, progesterone, and neurotransmitters

**Figure 3:**
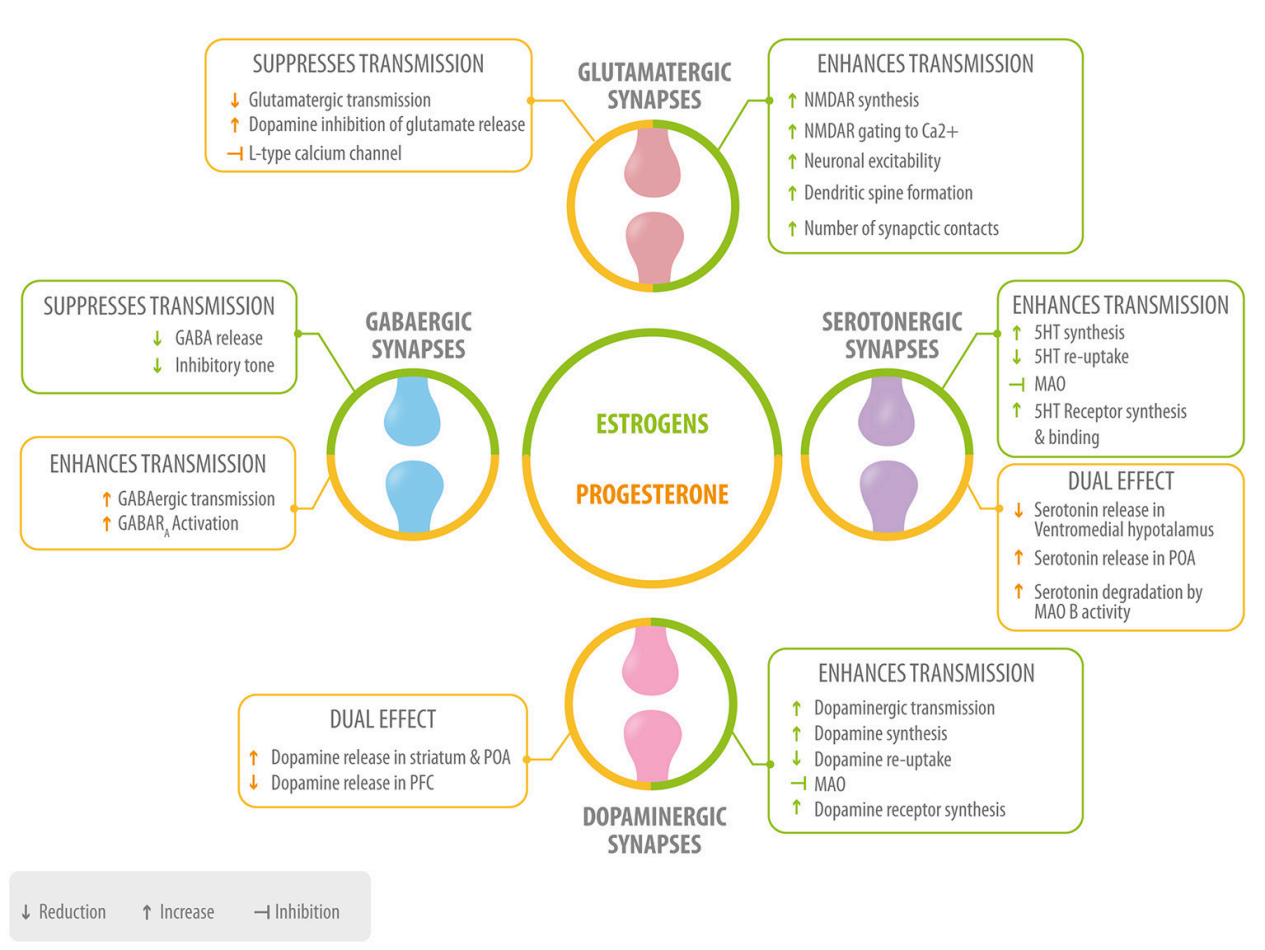
Role of neurosteroids in the modulation of the four main neurotransmitters. Estrogen (green) and progesterone (yellow) interact with GABAergic, glutamatergic, serotonergic, and dopaminergic synapses at different levels: neurotransmitter synthesis, release, degradation, and neurotransmitter receptor synthesis, activation or inhibition 5HT, serotonin; MAO, monoamino oxidase; POA, preoptic area; PFC, prefrontal cortex.

Neurosteroids participate in the regulation and modulation of neurotransmitter systems and neuronal excitability. We will briefly describe the main role of four main neurotransmitters: glutamate, gamma-Aminobutyric acid (GABA), serotonin (5-HT), and dopamine; as well as some mechanisms through which estradiol, progesterone, and their metabolites act at the synaptic level (Figure 3).

- Glutamate is the main excitatory neurotransmitter in the brain and glutamatergic synapses can be found from the prefrontal cortex to brainstem areas, striatum, nucleus accumbens, thalamus, hypothalamus, and hippocampus (64). It is involved in cognitive processes such as memory and learning (65).
- GABA is the most abundant inhibitory neurotransmitter in the brain (66). GABAergic synapses are found in the striatum, substantia nigra, brainstem, thalamus, hippocampus, basal ganglia, and cerebellum. Since it has a fundamental role in balancing brain cell activity, alterations in these pathways can cause anxiety. Inversely, the potentiation of its synapses causes anxiolysis, as in the case of benzodiazepines. GABA also contributes to motor control and diminishes neuronal firing rates in the CNS (67).
- Serotonin (5-HT) has an important role in the limbic system (68). Serotonergic pathways extend from the raphe nuclei to all areas of the forebrain including the frontal cortex, striatum, thalamus, amygdala, hypothalamus, and hippocampus (67). They are important contributors to a sense of well-being. Serotonergic pathways also modulate a wide range of autonomic functions (i.e., digestion), the sleep-wake cycle, sexual behavior, affections, mood, and cognitive functions (69, 70).
- Dopamine is known as the reward neurotransmitter, regulating pleasure, addiction, decision making, motivation, motor control (71), and learning (72). Dopaminergic areas include the ventral tegmental area, nucleus accumbens, hippocampus, amygdala, prefrontal cortex, substantia nigra, striatum, hypothalamus, and pituitary gland (73).

#### Estrogen in synaptic transmission

Estrogen potentiates release of glutamate and acts on postsynaptic membranes via the positive modulation of the ionotropic NMDA receptor which is related to synaptic plasticity, learning, and memory (74). Estrogen also increases the expression of this receptor and its sensitivity to glutamate, which then induces an increase of neuronal sensitivity to synaptic input through calcium influx (75). Hence, glutamatergic potentiation by estrogen leads to an increase in neuronal excitability. This has been presented as a mechanism through which estrogen generates morphological plasticity changes such as an increase in spine density in the hippocampus, amygdala, and prefrontal cortex (PFC). The plasticity changes have been associated with the role that estrogen has in improving learning, memory, and other cognitive functions (14).

Another effect of estrogen is to act as a suppressor of GABAergic transmission (76, 77) through the inhibition of the L-type Ca^2+^ channel required for GABA release in the presynaptic terminal (78). In the striatum and the PFC, the inhibition of GABAergic synapses promotes an increase in the synaptic transmission of glutamatergic and dopaminergic neurons (76, 78).

Serotonergic synapses are regulated by estrogen at different levels, including the promotion of serotonin synthesis through an increase in tryptophan hydroxylase enzyme levels (79). Additionally, estrogen inhibits serotonin degradation by monoamine oxidase, an enzyme with a central role in the catabolism of monoaminergic neurotransmitters (80), and inhibits serotonin reuptake from the synaptic cleft back to the presynaptic neuron (81), resulting in an increase in serotonin availability. Also, estrogen increases serotonin receptor levels via gene expression (82), and is reported to induce the increase of serotonin binding with the 5-HT receptor (69). The fact that serotonergic transmission in limbic areas and emotional functions is potentiated by estrogen, strongly suggests a role of the latter in mood and emotional states in women (83). Finally, estrogen increases dopamine synthesis and decreases its degradation, reuptake, and recapture. It also upregulates dopaminergic receptors (67, 84). The effect of estrogen on the dopaminergic system is of significant importance in the PFC, a region with high amounts of estrogen compared to other cortical areas (84, 85), where the presence of this neurosteroid impacts working memory function by affecting dopamine levels (84). Also, through these actions, estrogen protects certain cognitive functions in the presence of stress in menopausal women (86, 87). Furthermore, through its effects on PFC and limbic regions (such as the nucleus accumbens), estrogen influences emotional and motivational behaviors, for example by decreasing impulsive behaviors (21, 88).

#### Progesterone in synaptic transmission

Progesterone and allopregnanolone have an inhibitory role upon glutamatergic synapses (89). In the PFC, the progesterone metabolite allopregnanolone inhibits dopamine induced glutamate release (90), a mechanism that may be of special importance in relating the effects of progesterone to cognition and neuropsychiatric diseases (91). Allopregnanolone also inhibits glutamate release through an inhibition of the L-type Calcium channel (92). It has been observed that allopregnanolone, through the potentiation of GABAergic synapses, leads to a decrease in glutamate receptor efficiency (93, 94). Through some of these mechanisms, progesterone decreases neuronal excitability in glutamatergic projections.

Progesterone, especially allopregnanolone, potentiates GABAergic synapses through GABA A receptor activation, increasing the openings of GABA-gated chloride channels (95) and thus inhibiting synaptic transmission (93, 94). For this reason allopregnanolone has been attributed as having anti-anxiety effects similar to those of benzodiazepines and other positive modulators of GABA-A receptors (96, 97). Also, decreased circulating allopregnanolone levels are associated with depression in humans, and antidepressant treatment is associated with an increase in this metabolite (11, 98).

With regard to its possible effects on cognition, the interaction of progesterone with GABA receptors in the hippocampus could give a reasonable explanation for why exogenous administration of progestins has a negative impact on the performance of healthy women in working memory tests (99).

When administered after an estrogen dose, progesterone increases serotonergic neurotransmission in the preoptic area of the hypothalamus (POA) (100). However, in the ventromedial area of the hypothalamus, administration of estrogen, and progesterone reduces serotonin release (101).

Other studies show that progesterone decreases serotonergic neurotransmission by decreasing the expression of serotonin receptors (102) and increasing serotonin degradation through monoamine oxidase B (79). Furthermore, the stimulating effect of progesterone on serotonin release in POA could be associated with a decrease in copulatory behavior (103). Thus, the coordinated action of estrogen followed by progesterone, would enhance serotonergic synaptic activity, while isolated progesterone would inhibit it, depending on the brain region.

Progesterone, as well as allopregnanolone, interacts with dopaminergic systems (104, 105). In the striatum and POA, progesterone can stimulate dopamine release only if there has been a preexposure to estrogen (106, 107). In the striatum, this action could be associated with the observed improvement in sensorimotor functions during phases of the menstrual cycle when progesterone is elevated (108). In the POA this action may mediate some influence on maternal behavior (91).

In the nucleus accumbens, allopregnanolone has a largely positive modulatory effect over the release of dopamine (109), which could have an impact on behaviors that lead to drug abuse, including depression (91). In the PFC, allopregnanolone has an inhibitory effect on dopamine release (109), possibly related to the modulation of emotion during physiological and pathological conditions (108). In this way, the effect of progesterone on dopaminergic systems would depend primarily on the previous priming by estrogen and on the location of its activity.

## Effects of exogenous administration of synthetic steroid hormones in behavior, mood changes, and cognitive abilities

### Exogenous administration of synthetic steroids: contraceptives

There are millions of women who take hormonal contraceptives on a daily basis, often starting at a young age, a time when sex hormones have important organizational effects on brain structure (110). There are three main formulations for hormonal contraception: monophasic combination, multiphasic combination, and progestin-only formulations (Figures 1G,H). These can be administered either orally, by IM injections, subcutaneous implants, or through medicated intrauterine devices (IUDs).

Combined hormonal contraceptive pills by definition combine progestin with one of two types of estrogens, most commonly ethinyl estradiol, and less commonly ethinyl estradiol 3-methyl ether, otherwise known as mestranol. The term progestin refers to synthetic progesterone (111). It is this component of oral contraceptives (OCs) that, through its binding with PRs at different levels, suppresses ovulation (112, 113). In addition to its interaction with progesterone receptors, progestins can also interact with other steroid receptors such as the androgen receptor (AR), estrogen receptor, glucocorticoid receptor, and mineralocorticoid receptor, because all of these receptor proteins exhibit a structural similarity (114). Those progestins which exhibit relatively high affinity to the AR, generally belong to the first generation of synthetic progestins and are derived from testosterone (115). Among these are medroxyprogesterone acetate (MPA) and norethynodrel. Second generation progestins have high binding affinity for the AR, so they also have androgenic effects. In contrast, newer progestins have a strong progestational action and exert anti-estrogenic, antigonadotropic, and antimineralocorticoid effects with decreased androgenic activity (116). Among these are third-generation progestins: desogestrel or gestodene, derived from levonorgestrel (LNG). Finally, fourth generation progestins exhibit partial antiandrogenic activity (117) or even no activity via the AR (118). Among these are drospirenone, a spirolactone derivative, and dienogest, derived from a non-ethinylated progestin.

In a recent study, which included a total of 1.061.997 women and adolescents aged 15–34 years who were living in Denmark and had no prior depression diagnosis, an association was found between the use of hormonal contraception, a first diagnosis of depression and the subsequent use of antidepressants, especially among adolescents. All types of hormonal contraceptives had a statistically significant association with depression among adolescents, with levonorgestrel-only products exhibiting the higher incidence rate (119). Another publication of the same group has shown that OC users have an increase in the incidence of suicide attempts and actual suicide. Unlike other studies, this study included young women and found that the relative risk (RR) for suicide attempt varied by age. RR estimates were 2.06 (95% confidence interval [CI], 1.92–2.21) for women between 15 and 19 years, 1.61 (95% CI, 1.39–1.85) for the 20–24 years interval and 1.64 (95% CI, 1.14–2.36) for women between 25 and 33 years. It should be noted that adolescents were more sensitive than older women to the influence of hormonal contraception with regard to first suicide attempt (120).

The greater effect of exogenous hormones on mood and suicidal attempts among adolescents can partially be explained by the fact that this is a period of neuronal plasticity (10), that is, that hormone levels can induce changes in neurons and direct the architectural and structural functionality of the brain (121).

Two studies that have included a large number of long-term OC users (more than 3 years) showed that the percentage of women who reported experiencing depressive symptoms declined as the number of years of use increased. In the study done by Duke et al. the effect plateaued after 5 years (122). The authors suggest that there is a “survivor bias” whereby women who experienced deterioration in mood or depressive symptoms that they themselves associated with oral contraceptive use, were more likely to have stopped taking oral contraceptives. In the study done by Skovlund et al. the risk for first use of antidepressants increased with length of use, and peaked after 6 months, thereafter the risk decreased (120). This also suggests that women that present depressive symptoms or mood changes related to OC use will stop the use of these (120). Furthermore, longitudinal studies regarding the effect that long-term use of hormonal contraception may have upon mood, should consider the different types of OC's and the reason for which women used these, since it has been shown that women who use OC's for reasons other than contraception, were 1.32 (95% CI 1.07–1.62) times more likely to be depressed than women who used them for contraception (122).

Another study, using randomized, double-blind, placebo controlled trials in a group of 332 women, showed that continuous use of combined OC's (150 μg levonorgestrel and 30 mg of ethinyl estradiol) significantly decreased general well-being in healthy women (123). This same group showed, with the same cohort (124), that levonorgestrel-containing OCs had negative effects on sexual function in young women diminishing sexual desire, arousal, and pleasure. On the other hand, orgasm, concern, responsiveness, and self-image were not significantly affected by them. Similar results can be observed in the long-acting subdermal implant systems, which release a continuous dose of levonorgestrel for 5–7 years. This dose would have a suppressive effect on kisspeptin, GnRH, and LH, which would then lead to the inhibition of ovulation for prolonged periods of time (125). This results in a decrease in the levels of endogenous estradiol and progesterone (126). It has been reported that women users of LNG subdermal implants will develop mood disorders (127), and are more likely to report mood swings, nervousness, and depression than women using non-hormonal methods (128). With regard to medicated IUDs, their mechanism of action has not yet been clearly defined (129), but disruption of ovulation has been reported in several studies (130). This effect would lead to a hormonal imbalance which, added to the direct action LNG could have in brain cells, can explain mood disorders found in IUD users. The Danish study mentioned above showed that in the 15 to 34-year-old group a 3.1 RR of antidepressant use (95% 2.47–3.84) was found, this being the highest within the contraceptives studied (120).

Furthermore, administration of levonorgestrel (in doses as those found in emergency contraception) in primates during the follicular phase, can inhibit ovulation, as shown by the profound suppression of estradiol and the increase in cycle length from 32 to 52 days (131). Studies done in sterilized women also showed that ovulation is generally inhibited or that short luteal phases are observed when LNG is administered in the early or late follicular phase, but not when administered during the LH peak.

This disruption on endogenous estradiol and progesterone balance, added to a possible direct effect on brain cells, depends on the phase of the cycle during which it is administered and could affect mood in women using emergency contraception (131–133). Further studies are needed in order to elucidate the effects of LNG, administered as emergency contraception, on mood in women. These studies should consider the phase of the cycle, and the number of doses ingested.

These results can be explained, as mentioned earlier, through the multiple ways in which progesterone and estrogens can influence neural cells. For example, exogenous progestin increases levels of monoamine oxidase, which decreases serotonin concentrations and thus potentially produces depressive symptoms, irritability, and mood disorders (127–134). The estrogen and progesterone hormonal profile of OC users differs from that of naturally cycling women (Figure 1); endogenous levels are lowered and fluctuations are suppressed via negative feedback mechanisms (18). Also, the administration of exogenous estradiol causes an increase of hepatic sex hormone-binding globulin (SHBG), which translates into a decrease in the fraction of free estradiol, progesterone, and testosterone (135). Both the decrease in the bioavailability of these steroids and the lack of cyclicity could explain mood deterioration and changes in brain activity observed in women that use combined OCs (11).

In addition, the increase of serotonin release stimulated by progesterone at the level of the POA nucleus in the hypothalamus would result in a decrease of copulatory behaviors (136). Considering that the effect of progestins is similar to that of progesterone, this together with the increase of SHBG could explain the decrease in libido that has been reported with the use of OC's (124, 137).

The activity of progestins will differ depending on whether they are administered together with estrogen, administered after estrogen or administered without estrogen. Therefore, when analyzing steroid hormone activity, not only the available concentration and its form of administration must be taken into account, but the formulation to be administered (monophasic, multiphasic, or progestin alone) should be considered as well.

Also, not all progestins act in the same way. MPA used in injectable formulations deserves special attention, since, as described earlier, many neuroprotective effects of progesterone seem to be mediated by its conversion into neuroactive metabolites, such as DHP and allopregnanolone. It has been reported that MPA blocks the conversion of progesterone into allopregnanolone by inhibiting the ARK1C enzyme (138), possibly blocking progesterone's neuroprotective effects. This action should be taken into consideration when administering these kinds of preparations to women, since, and despite its benefits in the treatment of uterine bleeding, it may have serious deleterious effects on the myelination of neural cells.

Hence, when considering the effect that exogenous hormones exert over the body, their impact on the CNS and their influence on mood, behavior, and cognition should be evaluated.

### Exogenous administration of synthetic steroids: menopausal hormone therapy

Life expectancy has increased from approximately 50 to 80 years, but the age of menopause in women is relatively fixed. Given this, a larger number of women will spend over one-third of their lives in the postmenopausal state. Therefore, the repercussions of diminishing levels of estrogen on multiple aspects of women's health are relevant (139). Hormone therapy (HT) protocols for perimenopausal women, or those at an early postmenopause stage, have been developed to correct symptoms and prevent diseases related to the decline in hormonal secretion. HT may be administered in a continuously combined formulation (estrogen + progesterone), a sequentially combined formulation or, as an unopposed estrogen-only treatment (Figure 1I) (140, 141). Nowadays FDA approved indications for HT include vasomotor symptoms, prevention of bone loss, hypoestrogenism and the genitourinary syndrome of menopause/vulvo-vaginal atrophy (32, 142). Also, depression and cognitive impairment are associated with declining steroidal hormone levels, effects that can be reverted with proper HT (33, 135). However, HT prescription may be associated with a higher incidence of estrogen-sensitive cancers, heart disease, stroke, and venous thromboembolism. Thus, it is appropriate to keep this under consideration when counseling menopausal women (32).

Goodnick et al. (143) demonstrated that women undergoing HT exhibit a reduction in depressive symptoms (143). Other studies have shown an improvement in verbal memory, attention, and reasoning (140) together with a reduction in the risk of dementia (144). These results may be explained by estrogen's neuroprotective capacity combined with its role in synaptic systems that influence memory and cognition. Estrogens also have a neurotrophic action, as demonstrated by an increase in the number of dendritic spine formations in the amygdala, hippocampus, and the PFC (144, 145). Collectively, functional magnetic resonance studies have shown that postmenopausal women under HT have greater activation, larger volume, and increased cerebral blood flow to the hippocampus (an area known to be affected in major depressive disorder) compared with non-users of HT (146).

HT will have pleiotropic effects that will vary according to the timing of initiation, the form of estrogen and of progestin used, the route of administration (e.g., oral vs. transdermal) and the therapeutic scheme used (e.g., continuous or sequential).

Regarding time of initiation, a critical period or window of opportunity hypothesis has shown that neurons become insensitive to estrogens after long-term hormone deprivation (87, 147). According to this hypothesis, only the administration of estrogens during a critical period related to the cessation of ovarian function will render beneficial effects. Estrogens given after this critical period could cause negative effects (148). A possible explanation implicates the loss of the alpha estrogen receptor (ERα) in the brain resulting from long-term hormone deprivation. This would not permit estrogen to act via its classical and non-classical pathways (14). Thus, during a critical period following the cessation of ovarian function, the presence or absence of estrogen permanently alters the CNS. The end result is that the presence of estrogen during the critical period has beneficial effects on brain function, and decreases the risk of neurodegeneration and cognitive impairment (140, 144).

With regard to the form of estrogens and progestins administered, the proportion of estrogenic forms differs during menopause compared to premenopause. Initially, the proportion of estradiol (E2) is approximately five times greater than that of estrone (E1) (141). Later, during menopause and postmenopause, E1 is the predominant form of estrogen. Thus, to mimic premenopausal physiology it is necessary to administer E2 in HT (83). The transdermal use of E2 has shown a consistently positive impact on women's mood during their perimenopausal stage (149, 150). Conversely, the administration of HT in the form of continuous conjugated equine estrogens (CEEs) (0.625 mg/d), rich in E1, does not show positive results with regard to mood (151).

When combined HT is indicated, it is important to consider the type of progestin utilized. As mentioned earlier, multiple studies have shown negative effects on the myelination of neural cells with MPA use (87, 152).

A clear example of the importance of considering the timing and the type of estrogen and progestin administered is shown in the large multicenter WHI (Women's Health Initiative) randomized placebo-controlled trial (24, 152–155). According to the WHI study, women with uterus received a combined HT consisting of MPA (2.5 mg/d; Prempro) and CEE (0.625 mg/d) (24, 154, 155) and women without a uterus received CEE only (152, 153). However, more than 65% of the population of this study was older than 60 years of age, meaning that many of the women included in it were beyond the time when they could benefit from HT. After an average follow-up of 4–5 years, the WHIMS (Memory Study portion) failed to observe any improvement in measures of global cognition or rates of either mild cognitive impairment or dementia in women taking combined HT compared with placebo (24, 155). Furthermore, WHIMS observed an increased risk of dementia with this HT (155). These results can be partly explained by the lack of positive results that could endorse the use of CEE in postmenopausal women, the known adverse effects that MPA has upon myelination and the negative effects of estrogen administration out of the critical opportunity period.

## Conclusions and future directions

**Figure 4:**
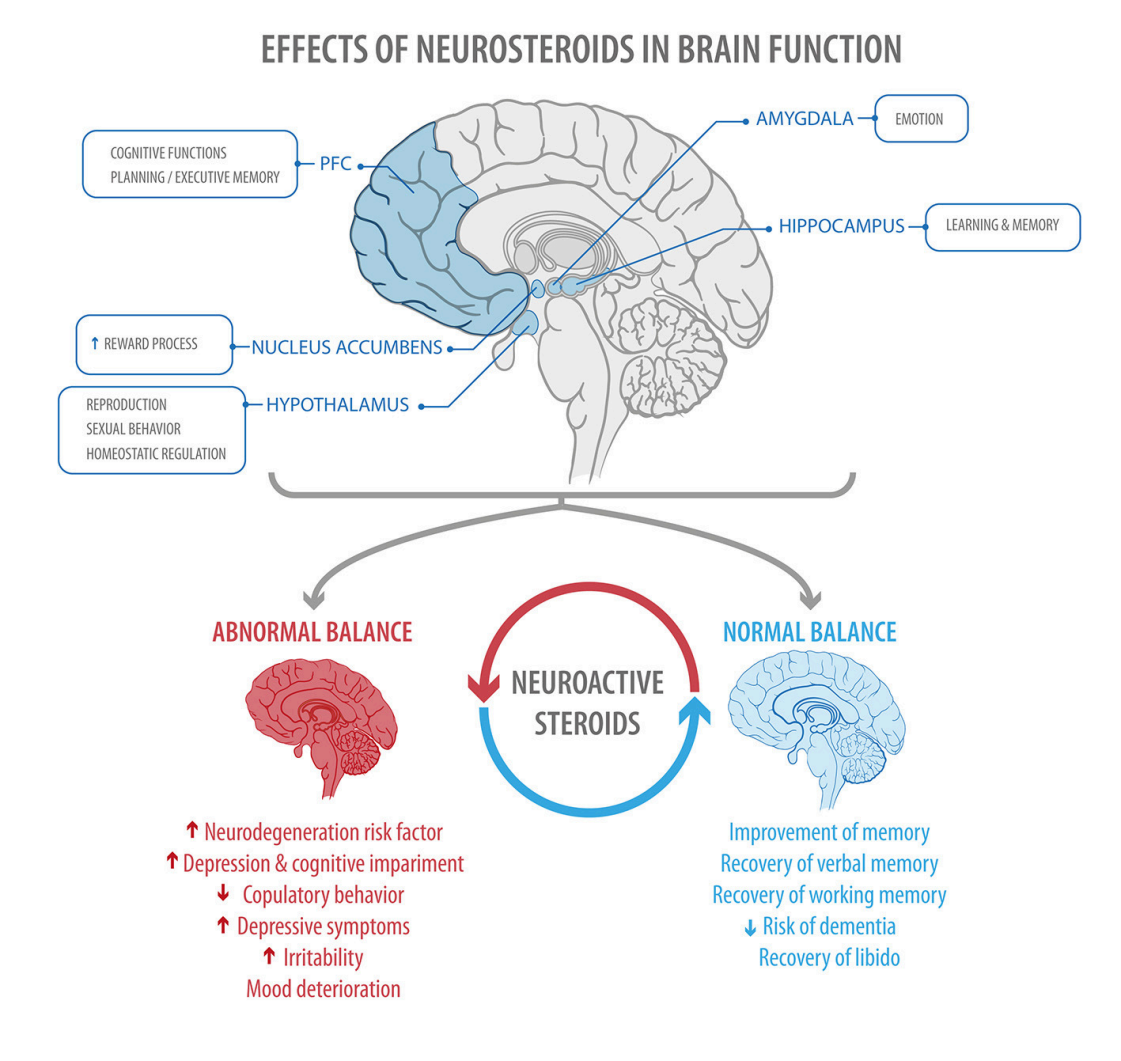
The figure shows areas of the brain regulated by steroid hormones **(Top)**, and some of the effects found when a normal or abnormal balance between estrogen and progesterone is present **(Bottom)** PFC, prefrontal cortex.

The present review shows that fluctuations in steroid hormones, influenced by factors such as age and health status, have consequences at the level of CNS and PNS. Utilizing both classical and non-classical pathways, neurosteroids participate in the physiological regulation of neurogenesis, neuronal survival, synaptic function, and myelin formation, thus influencing neuronal plasticity. Because of these effects, neurosteroids will have different modulatory actions, exerting control over mood, cognition, and behavior. Additionally, they have a neuroprotective role in relation to certain neurocognitive pathologies (Figure 4).

This must be taken into consideration when treating patients with pathologies that affect ovarian function, such as PCOS, hyperprolactinemia, or hypothalamic anovulation, among others; and also when a woman consults for changes in mood or cognition.

On the other hand, hormone therapy during menopause and hormonal contraceptives are two modes of treatment through which exogenous steroids are administered to women. When facing a need for the administration of exogenous hormones, the stage of life each woman finds herself in should always be considered. When treating adolescents, special care should be taken due to the temporal plasticity window. For example, there are conditions in young women, such as anorexia nervosa, during which the levels of estrogen and progesterone will be low. In these cases, it is necessary, as part of the treatment, to administer hormones. The same can be said of conditions such as aging, during which steroidal hormone decline has been shown to have negative effects. Thus, through the scientific evidence analyzed in this review, it should be clear that, when an exogenous steroid therapy is indicated, the timeliness of its administration and the types of estrogen and progestin utilized must be precisely taken into account.

Finally, questions to consider in future investigations include: (i) in terms of the ovarian continuum, what patterns of ovarian activity will have negative effects on the nervous system, and what patterns will have positive effects? (ii) Should the effects of OCs on the CNS be considered as adverse? Could they have positive effects? (iii) Is there a different effect on the brain when OCs are taken during adolescence? What is the effect of emergency contraception upon the adolescent brain? (iv) To what degree should HT formulations be guided by physiological patterns of exposure? (i.e., cyclical vs. continuous).

In summary, the activity exerted by steroid hormones on the nervous system emphasizes the notion that achieving hormonal balance is a useful tool in seeking the well-being of women. Healthcare providers, as well as the general population, should be aware of this knowledge.

## Author contributions

JD and MA researcher and writer. NM assistant researcher. FS assistant researcher, image design manager. SM writer and proofreader. PV corresponding author, tutor research guide, writer and proofreader.

### Conflict of interest statement

The authors declare that the research was conducted in the absence of any commercial or financial relationships that could be construed as a potential conflict of interest. The handling editor is currently co-organizing a Research Topic with one of the authors PV, and confirms the absence of any other collaboration.
